# Brentuximab Vedotin in Advanced-Stage Mycosis Fungoides/Sézary Syndrome with Low CD30 Expression: Real-World Data from the German Cutaneous Lymphoma Network

**DOI:** 10.3390/cancers18010097

**Published:** 2025-12-28

**Authors:** Christoph Blazejak, Mathias Oymanns, René Stranzenbach, Uwe Hillen, Christina Mitteldorf, Jan P. Nicolay, Marion Wobser, Philipp Schrüfer, Janika Gosmann, Ulrike Wehkamp, Nina Booken, Alexander Kreuter, Edgar Dippel, Claus-Detlev Klemke, Maria Weyermann, Rudolf Stadler, Chalid Assaf

**Affiliations:** 1 Department of Dermatology, Helios Klinikum Krefeld, Health and Medical University Düsseldorf-Krefeld, 47805 Krefeld, Germany; 2 Department of Dermatology, Universitätsklinikum Bochum, 44801 Bochum, Germany; 3 Dermatology Practice Dr. R. Stranzenbach, 58285 Gevelsberg, Germany; 4 Department of Dermatology, Vivantes Klinikum Neukölln, 12351 Berlin, Germany; 5 MVZ Dermatopathologie Duisburg Essen GmbH, 45147 Essen, Germany; 6 Department of Dermatology, Venereology and Allergology, University Medical Center Göttingen, 37075 Göttingen, Germany; 7 Department of Dermatology, University Medical Center Mannheim, 68167 Mannheim, Germany; jan.nicolay@umm.de; 8 Department of Dermatology, Venereology and Allergology, University Hospital Würzburg, 97080 Würzburg, Germanyphilipp.schruefer@hin.ch (P.S.); 9 Skinmed AG, 5600 Lenzburg, Switzerland; 10 Department of Dermatology, Venereology, Allergology and Phlebology, Johannes Wesling Clinic, University Hospital of the Ruhr University Bochum, 32429 Minden, Germany; janika.gosmann@unimedizin-ffm.de (J.G.);; 11 Department of Dermatology and Allergy, University Hospital Schleswig-Holstein, 24105 Kiel, Germany; 12 Medical School Hamburg, University of Applied Sciences and Medical University, 20457 Hamburg, Germany; 13 Helios Kliniken GmbH, 10117 Berlin, Germany; 14 Department of Dermatology and Venereology, University Medical Center Hamburg-Eppendorf, 20246 Hamburg, Germany; 15 Department of Dermatology, Helios Klinikum Oberhausen, Universität Witten/Herdecke, 46045 Oberhausen, Germany; 16 Department of Dermatology and Venereology, Klinikum Ludwigshafen, 67063 Ludwigshafen, Germany; 17 Department of Dermatology and Skin Tumor Center, Municipal Hospital Karlsruhe, Academic Teaching Hospital of the University of Freiburg, 76133 Karlsruhe, Germany; 18 Department of Health Care, Niederrhein University of Applied Sciences, 47805 Krefeld, Germany; maria.weyermann@hs-niederrhein.de; 19 Institute for Molecular Medicine, Medical School Hamburg, University of Applied Sciences and Medical University, 20457 Hamburg, Germany; 20 Faculty of Medicine, Health and Medical University Düsseldorf-Krefeld, 47805 Krefeld, Germany

**Keywords:** cutaneous T-cell lymphoma, CTCL, Sézary syndrome, mycosis fungoides, brentuximab vedotin, CD30, treatment

## Abstract

**Background/Objectives**: Advanced-stage mycosis fungoides (MF) and Sézary syndrome (SS) are aggressive forms of cutaneous T-cell lymphoma (CTCL) for which treatment options are limited and prognosis is poor. Brentuximab vedotin (BV), an anti-CD30 antibody–drug conjugate, has demonstrated high response rates in patients with CD30 expression ≥ 10%. However, data on its efficacy in cases with low CD30 expression (<10%) remain scarce. **Methods**: This retrospective analysis evaluated the real-world efficacy of BV in patients with advanced-stage MF/SS and low CD30 expression. A retrospective analysis was conducted on 32 patients across 11 German CTCL expert centers. All patients had advanced-stage MF or SS with CD30 expression < 10% and received BV at the standard dose. Treatment response was assessed using EORTC-ISCL criteria. **Results**: All patients had received prior systemic therapies (median: 3) with 36% having undergone prior mono- or polychemotherapy. The study population included 30 MF (stage IIB) and two SS cases. The overall response rate (ORR) in this population was 53.1% (17/32). A complete response (CR) was achieved in 12.5% (4/32), a partial response (PR) was achieved in 40.6% (13/32), stable disease (SD) was seen in 18.8% (6/32), and progressive disease (PD) was seen in 28.1% (9/32). The median progression-free survival (PFS) was 4.0 months (arithmetic mean: 6.38; range: 0.5–15.5), and the median time to next treatment (TTNT) was 7.25 months (arithmetic mean: 7.30; range: 2.00–15.5). **Conclusions**: BV demonstrated encouraging activity in heavily pretreated advanced MF/SS with low CD30 expression, achieving an ORR comparable to that observed in patients with higher CD30 levels. While response rates were similar, PFS was shorter. These findings suggest that BV remains a potential therapeutic option in this patient population and merits further prospective investigation.

## Simple Summary

Advanced-stage mycosis fungoides (MF) and Sézary syndrome (SS) are aggressive cutaneous T-cell lymphomas with limited treatment options and poor prognosis. Brentuximab vedotin (BV), an anti-CD30 antibody–drug conjugate, has shown high efficacy in patients with CD30 expression ≥ 10%, but data in low-CD30-expressing tumors are limited. This retrospective multicenter study evaluated 32 patients with advanced MF/SS and CD30 expression < 10% treated with standard-dose BV. All patients had received prior systemic therapies (median: 3). The overall response rate (ORR) was 53.1%, including 12.5% complete and 40.6% partial responses. Median progression-free survival (PFS) was 4.0 months, and median time to next treatment (TTNT) was 7.3 months. These results indicate that BV can induce meaningful responses even in tumors with low CD30 expression. Our findings support the use of BV as a therapeutic option in this population and highlight the need for further prospective studies to optimize patient selection and treatment outcomes.

## 1. Introduction

Primary cutaneous T-cell lymphomas (CTCL) comprise a heterogeneous group of non-Hodgkin lymphomas characterized by the infiltration of malignant T cells into the skin, with potential progression to extracutaneous organs in advanced stages, but without systemic manifestation at the time of diagnosis [1]. Mycosis fungoides (MF) is the most common subtype, typically exhibiting an indolent course in the early stages of the disease. Five-year survival exceeds 90% in stage IA MF but declines profoundly to approximately one-third once extracutaneous dissemination occurs [2,3,4]. CTCL remains relatively rare compared with systemic lymphomas, with an estimated incidence of ~1 per 100,000 persons and a median age at diagnosis of 55–60 years [5,6,7].

The management of early-stage CTCL (≤stage IIA) focuses on skin-directed therapies—including topical corticosteroids, phototherapy (PUVA), topical chlormethine, and immunomodulatory or retinoid-based approaches—which yield high response rates with acceptable toxicity [8,9,10,11]. In contrast, advanced-stage disease often requires systemic therapy. However, traditional cytotoxic agents such as gemcitabine or doxorubicin frequently achieve only transient remissions and are associated with cumulative toxicity [12,13,14,15,16].

Brentuximab vedotin (BV), an antibody–drug conjugate that targets CD30 [17], represents an important therapeutic advance for CTCL. BV has demonstrated meaningful and durable clinical activity and is now widely used in advanced MF and primary cutaneous anaplastic large cell lymphoma (pcALCL). Although BV was approved based on the pivotal phase III ALCANZA trial—which required ≥10% CD30 expression in at least one biopsy for enrollment—responses were observed across a broad spectrum of CD30 expression levels. The study reported an overall response rate (ORR) of 67.1% and an objective response rate lasting ≥4 months (ORR4) of 56.3% in the combined MF/pcALCL cohort [18,19,20].

Subsequent real-world analyses and case series have further documented objective responses, including complete remissions, in patients with CD30 expression < 10%, supporting the notion that BV may be effective against tumors with low or heterogeneous CD30 expression [21,22,23,24]. Nevertheless, clinical data specifically addressing outcomes in advanced MF or Sézary syndrome (SS) with CD30 expression < 10% or even <5% remain limited.

To address this clinically relevant question, we conducted a retrospective study within the German Cutaneous Lymphoma Network to characterize treatment patterns and the duration of benefit from BV in patients with advanced-stage MF/SS and low CD30 expression (5–9% and <5%).

## 2. Materials and Methods

### 2.1. Patients

Between 2014 and 2020, 32 patients from 11 centers participating in the German Network for Cutaneous Lymphomas received BV monotherapy. Eligible patients had a histologically confirmed diagnosis of advanced CD30^+^-CTCL according to the WHO–EORTC classification and demonstrated <10% CD30 expression by immunohistochemistry using routinely the anti-CD30 antibody Ber-H2 (Dako, Agilent Technologies, Santa Clara, CA, USA). Clinical and demographic data were collected retrospectively from institutional databases.

The study protocol was approved by the Ethics Committee of Nordrhein (No. 242/2020) and conducted in accordance with the Declaration of Helsinki. A waiver of informed consent was granted due to the retrospective design.

### 2.2. Treatment and Assessments

BV was administered according to institutional standards. Disease burden was assessed at baseline and at the end of treatment by complete physical examination, including full-body skin evaluation with quantification of tumor burden, routine laboratory studies, and cross-sectional imaging (contrast-enhanced computed tomography of the chest, abdomen, and pelvis), consistent with the assessment schedule used in the ALCANZA trial.

Treatment response and progression were evaluated according to the ISCL/EORTC consensus criteria, incorporating assessment of the skin, lymph nodes, blood, and visceral involvement [25]. Responses were categorized as complete response (CR), partial response (PR), stable disease (SD), or progressive disease (PD). For patients achieving an objective response (CR + PR), progression-free survival (PFS) and time to next treatment (TTNT) [26] were calculated.

### 2.3. Safety

Adverse events (AEs) were graded using the National Cancer Institute Common Terminology Criteria for Adverse Events (CTCAE), according to the version applicable at the time of treatment, from grade 0 (none) to grade 4 (life-threating or fatal). AE evaluation included clinical examination and standard laboratory testing. Previous and subsequent systemic therapies were recorded for all patients.

### 2.4. Statistical Analysis

Descriptive statistics were used to summarize baseline demographic and clinical characteristics, treatment history, response rates, and adverse events. Categorical variables were compared using the Mantel–Haenszel χ^2^ test; when expected cell counts were <5, Fisher’s exact test (two-sided) was applied. Continuous variables, including age at diagnosis, age at treatment initiation, and duration of treatment, were compared across groups using the Kruskal–Wallis test.

PFS and TTNT were calculated from the start of BV to the date of documented progression, initiation of subsequent therapy, or death, as appropriate. Patients without an event were censored at the date of last follow-up. Survival functions were estimated using the Kaplan–Meier method.

All statistical analyses were performed using SAS software, version 9.4 (SAS Institute Inc., Cary, NC, USA). Kaplan–Meier survival curves were generated using SPSS Statistics, version 25 (IBM Corp., Armonk, NY, USA). A two-sided *p*-value < 0.05 was considered statistically significant.

## 3. Results

### 3.1. Patient Characteristics

Thirty-two patients were included, of whom 30 (93.8%) had mycosis fungoides and 2 (6.3%) had Sézary syndrome. The cohort comprised 21 men (68.8%) and 10 women (31.3%), with a mean age of 60.3 years (range, 27–88 years).

30 patients had stage MF IIB disease with 2/30 having a large cell transformation according to the WHO–EORTC classification and had received prior CTCL-directed therapy. The median number of previous systemic or skin-directed treatments was 3 (range, 1–7). Of these, 8/22 (36%) had received prior monochemotherapy, mostly gemcitabine, pegylated doxorubicin, or doxorubicin-based polychemotherapy (e.g., CHOP or CHOEP).

In our cohort, all cases showed CD30-expression and cases were categorized into two predefined groups: <5% CD30 expression and 5–9% CD30 expression. The majority of patients (62.5%) fell into the <5% group, while 37.5% demonstrated CD30 expression between 5% and 9%. We did not implement further stratification of CD30 expression levels due to the well-recognized inter- and intra-observer variability associated with microscopic quantification, which limits the reliability of more granular percentage thresholds (Table 1).

### 3.2. Treatment Outcomes

**Figure 1:**
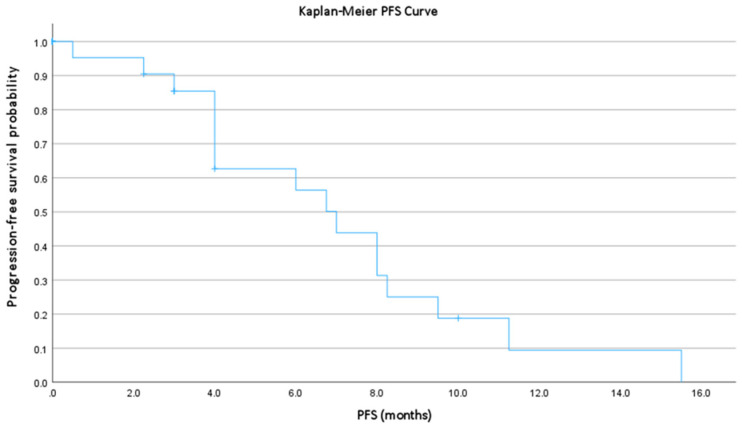
Progression-free survival in advanced CTCL patients with CD30 <10% treated with brentuximab vedotin. Kaplan–Meier curve demonstrating PFS in 28 patients with advanced MF/SS and CD30 expression < 10%. n = 21 patients with PFS > 0. Median PFS was 4.0 months (range, 0.5–15.5).

The overall response rate (ORR) was 53.1% (17/32), including 4 complete responses (CR, 12.5%) and 13 partial responses (PR, 40.6%). Stable disease (SD) was observed in 6 patients (18.8%), and progressive disease (PD) in 9 patients (28.1%). Median PFS was 4.0 months (range, 0.5–15.5 months; Figure 1), cases in which complete remission was achieved were included in the analysis as censored observations. Cases were excluded due to missing data (n = 4) or due to PFS = 0 (n = 7). Median TTNT was 7.3 months (range, 2.0–15.5 months; Table 2).

Treatment response by CD30 expression subgroup: responses were observed in both CD30 subgroups. Patients with CD30 <5% achieved objective responses, supporting the clinical activity of BV even at very low CD30 expression. No statistically significant differences were identified in ORR between subgroups, although sample size limits inferential power (Table 3).

### 3.3. Adverse Events

Therapy with BV was generally well tolerated across all patients. Reported adverse events (AEs) included peripheral neuropathy grade 1–2 in 7 patients (6 grade 1, 1 grade 2), maculopapular exanthema in 2 patients (6%), leukopenia in 2 patients (grade 1, 6%), one case of hepatitis (grade 3, 3%) and one case of pancreatitis (grade 3, 3%).

## 4. Discussion

CTCLs represent a heterogeneous group of non-Hodgkin lymphomas that primarily affect the skin. These malignancies express a wide range of surface antigens, including CD30, a member of the tumor necrosis factor (TNF) receptor superfamily, which has emerged as a clinically relevant biomarker and therapeutic target [27,28].

CD30 expression in CTCL is usually inconsistent and frequently patchy within individual lesions, being most pronounced in advanced or transformed MF and in pcALCL [29,30]. Physiologically, CD30 is expressed on activated T cells and is transiently induced during immune activation. Its sustained or aberrant expression in CTCL has been linked to disease progression, large-cell transformation, and resistance to apoptosis [31]. While not all CTCL lesions express CD30, even partial expression can have clinical significance [32]. Importantly, antibody–drug conjugates (ADCs) targeting CD30 can exert cytotoxic effects on Neighboring CD30-negative tumor cells through a bystander effect, whereby the cytotoxic payload diffuses to adjacent CD30-negative tumor cells [33].

BV is a CD30-directed ADC consisting of a chimeric anti-CD30 monoclonal antibody conjugated to monomethyl auristatin E (MMAE), a potent microtubule-disrupting agent. Upon binding to CD30-positive cells, BV is internalized and MMAE is released intracellularly, leading to cell-cycle arrest and apoptosis [34,35]. The pivotal ALCANZA phase III trial demonstrated significantly superior efficacy of BV over physician’s choice (methotrexate or bexarotene) in patients with CD30-positive CTCL, including MF and pcALCL, with higher ORR4 and improved quality of life. These results established BV as a standard treatment option and led to regulatory approval in both the United States and Europe. In a subgroup of tumor-stage MF (n = 19), the overall response rate reached 68%, including 16% complete responses, highlighting the potent activity of BV in this subset.

Interestingly, benefit from BV extends to patients with low CD30 expression. Although the ALCANZA trial required ≥10% CD30 positivity, durable responses were observed across the entire expression spectrum in subsequent real-world studies, including cases with <5% expression [21,22]. This challenges the idea that CD30 abundance is strictly necessary and suggests that limited expression may be sufficient for ADC uptake. Additionally, other mechanisms, such as the bystander effect or immune-mediated modulation, may contribute to the observed clinical efficacy.

Retrospective analyses of ALCANZA and multiple real-world studies have suggested a trend toward higher response rates and longer PFS in patients with strong CD30 expression (>50%) [18,19]. However, meaningful and durable responses have also been reported in patients with lower or heterogeneous expression (10–30%), and no validated quantitative threshold beyond the 10% cut-off has been defined [21,22]. These findings indicate that, while CD30 is a useful biomarker for target engagement, it is not sufficient to predict treatment response or survival outcomes.

The clinical interpretation of CD30 expression is further complicated by technical and biological variability. Immunohistochemical (IHC) detection is influenced by pre-analytical factors, antibody selection, staining protocols, and scoring methods [36]. Moreover, intralesional heterogeneity and temporal fluctuations in CD30 expression, potentially induced by prior therapies, further limit reproducibility [37]. These inconsistencies make it difficult to establish a universal threshold and emphasize the need for standardized IHC quantification and digital image analysis to improve diagnostic accuracy and reproducibility.

In our cohort, the ORR of 53.1%, including 12.5% complete and 40.6% partial responses, is consistent with previously reported BV efficacy in relapsed or refractory CTCL, even though most patients had received multiple prior systemic therapies. Reported ORRs for BV typically range from 40% to 60%, confirming its reproducible clinical activity across diverse populations. The median PFS (4.0 months) and median TTNT (7.3 months) observed in our heavily pretreated cohort of advanced stage CTCL-patients are comparable to published data, indicating durable disease control in a subset of patients. Notably, objective responses occurred irrespective of CD30 expression level, including in patients with <5% expression, reinforcing the emerging view that BV maintains activity even in minimally CD30-expressing disease. The absence of a statistically significant difference in ORR between CD30 subgroups likely reflects sample size limitations and the exploratory nature of this analysis.

The observation of responses in tumors with low or absent CD30 expression is biologically intriguing and has been increasingly recognized across lymphoma subtypes (Table 4) [3,19,38,39]. Several non–mutually exclusive mechanisms may underlie this phenomenon. First, standard IHC techniques may underestimate CD30 expression, as antigen density can be below detection thresholds or unevenly distributed within tumor tissue, with interlesional and intraindividual heterogeneity [37,40]. Second, the bystander effect allows MMAE released from lysed CD30-positive cells to diffuse locally and kill adjacent CD30-low or -negative cells, extending the cytotoxic reach beyond the antigen-expressing compartment. Third, BV may exert immunomodulatory effects within the tumor microenvironment (TME), including the depletion of CD30-expressing regulatory T cells (Tregs) and tumor-associated macrophages (TAMs) [41,42], which could restore effector T-cell activity and enhance antitumor immunity. These immune-mediated mechanisms are particularly relevant in CTCL, where the immune microenvironment plays a central role in disease persistence and progression [43,44].

Future research should focus on refining biomarker-driven patient selection and exploring strategies to enhance response durability. The integration of digital pathology, multiplex immunohistochemistry, and RNA-based profiling could enable a more accurate and dynamic assessment of CD30 and related immunologic markers [45,46]. Furthermore, rational combination approaches are under active investigation. Early-phase trials are evaluating BV in combination with radiotherapy [47,48], immune checkpoint inhibitors, and histone deacetylase inhibitors (HDACi) [49,50], aiming to increase response rates synergistically and overcome acquired resistance. Adding BV to these regimens may increase both direct cytotoxicity and immune reactivation, providing a multi-pronged therapeutic approach.

Overall, our findings reinforce the robust and reproducible efficacy of BV in relapsed or refractory CTCL, regardless of CD30 expression level. The data support the concept that CD30 expression exists on a continuum, where even low or heterogeneous expression can yield clinically meaningful benefit. While higher CD30 expression may correlate with longer PFS and greater depth of response, the relationship between antigen density and therapeutic efficacy appears to be non-linear and context dependent.

## 5. Conclusions

In conclusion, CD30 remains a key biological and therapeutic marker in CTCL; however, its quantitative assessment alone is insufficient for optimal patient stratification. Treatment decisions should therefore incorporate CD30 status alongside disease stage, prior therapy, and comorbidities. Continued efforts to refine biomarker precision and develop BV-based combination regimens are essential to optimize patient outcomes. These insights highlight the evolving paradigm in CTCL management, in which targeted therapies such as BV are increasingly guided by molecular context rather than rigid biomarker thresholds.

## Figures and Tables

**Table 1:** Baseline patient and disease characteristics (n = 32).

Characteristic	n (%)/Median (Range)
**Diagnosis**	
Mycosis fungoides	30 (93.8)
Sézary syndrome	2 (6.3)
**Sex**	
Male	22 (68.8)
Female	10 (31.3)
**Age (years)**	
Mean ± SD	60.3 ± 14.7
Median (Range)	63 (27–88)
**Disease stage**	
MF IIB	30 (93.7)
SS	2 (6.3)
**Prior therapies**	
Median number of prior systemic therapies	3
Range	1–7
Skin-directed therapies *	32 (100)
Systemic therapies ^†^	32 (100)
**CD30 expression**	
<5%	20 (62.5)
5–9%	12 (37.5)

* Skin directed therapies included, e.g., phototherapy, topical steroids. ^†^ Systemic therapies included interferon-a, bexarotene, MTX, mono- and polychemotherapies (36% had received prior mono- or polychemotherapy).

**Table 2:** Treatment response and survival outcomes (n = 32).

Outcome	n (%)/Median (Range)
**Best overall response**	
Complete response (CR)	4 (12.5)
Partial response (PR)	13 (40.6)
Objective response rate (ORR = CR + PR)	**17 (53.1)**
Stable disease (SD)	6 (18.8)
Progressive disease (PD)	9 (28.1)
Progression-free survival (PFS) *	
Median PFS (months)	4.0
Range	0.5–15.5
Time to next treatment (TNT) **	
Median TNT (months)	7.3
Range	2.0–15.5

* PFS calculated from initiation of brentuximab vedotin to progression or death; patients censored at last follow-up. ** TNT computed from initiation of brentuximab vedotin to initiation of subsequent systemic therapy.

**Table 3:** Treatment response by CD30 expression subgroup.

Parameter	CD30 <5% (n = 20)	CD30 5–9% (n = 12)	Total (N = 32)
Best overall response			
Complete response (CR)	2 (50%)	2 (50%)	4 (12.5%)
Partial response (PR)	9 (69.2%)	4 (30.8%)	13 (40.6%)
Objective response rate (ORR)	11/20 (64.7%)	6/12 (50%)	17 (53.1%)
Stable disease (SD)	3 (60%)	2 (40%)	6 (18.8%)
Progressive disease (PD)	6 (66.7%)	3 (33.3%)	9 (28.1%)

Abbreviations: ORR, objective response rate. Values shown as n (%), median (range).

**Table 4:** Correlation of CD30 Expression with BV Response in CTCL.

Study	Design/N	Population	CD30 Assessment	Correlation Tested	Key Findings
Kim et al., 2015 Phase II IIT [3]	Prospective, single-arm; ~32 pts	MF/SS stages IB through IVB at least one systemic therapy failure, broad CD30 range	IHC; multispectral imaging	CD30% vs. ORR (cutoff ~5%)	Higher responses when ≥5% CD30; responses still present <5%; multispectral often found CD30 when IHC “negative.”
Kim et al., 2021 ALCANZA Biomarker Sub-analysis [19]	Randomized Phase III; MF ± LCT subset, 131 pts	CD30 + MF (no further information about stages) ± LCT, ≥10% CD30 entry, but lesions variable	Central IHC on ≥2 biopsies	“CD30 min < 10%” vs. “CD30 min ≥ 10%”	BV benefit in both strata; no predictive threshold; inter-lesion variability.
Huen et al., 2018 pooled Phase II [38]	Pooled analysis of early trials; ~70 pts	Relapsed/refractory MF/SS, 19% stage IB/IIA, 81% advanced stage (IIB, IV), 53% with LCT	IHC	>5% vs. ≤5%	Trend to higher ORR > 5% (not significant); responses even ≤5%.
Jagadeesh et al., 2022 Pooled NHL analysis (CTCL subgroup) [39]	Cross-trial pooled; CTCL subset, 275 pts	CD30+ lymphomas, including CTCL. No further information on disease stage in CTCL is available	IHC	≥10% vs. <10%	No significant ORR difference; no predictive threshold.
Papadavid et al., 2021 EORTC real-world [22]	Retrospective, multicenter; 72 pts	Mostly (71/72 patients) advanced stage MF/SS (Stage IIB to IVB). Patients received at least one systemic therapy. 34/72 patients heavily pretreated (≥3 therapies)	IHC	CD30% as continuous & grouped	Responses across CD30% levels; not powered for cutoffs; supports no strict minimum.

## Data Availability

The data presented in this study are available on request from the corresponding author.

## References

[1] Willemze R., Cerroni L., Kempf W., Berti E., Facchetti F., Swerdlow S.H., Jaffe E.S. (2019). The 2018 update of the WHO-EORTC classification for primary cutaneous lymphomas. Blood.

[2] Agar N.S., Wedgeworth E., Crichton S., Mitchell T.J., Cox M., Ferreira S., Robson A., Calonje E., Stefanato C.M., Wain E.M. (2010). Survival Outcomes and Prognostic Factors in Mycosis Fungoides/Sézary Syndrome: Validation of the Revised International Society for Cutaneous Lymphomas/European Organisation for Research and Treatment of Cancer Staging Proposal. J. Clin. Oncol..

[3] Kim Y.H., Tavallaee M., Sundram U., Salva K.A., Wood G.S., Li S., Rozati S., Nagpal S., Krathen M., Reddy S. (2015). Phase II Investigator-Initiated Study of Brentuximab Vedotin in Mycosis Fungoides and Sézary Syndrome With Variable CD30 Expression Level: A Multi-Institution Collaborative Project. J. Clin. Oncol..

[4] Scarisbrick J.J., Quaglino P., Whittaker S., Bagot M., Guenova E., Papadavid E., Prince H.M., Sanches J.A., Miyashiro D.R., Servitje O. (2025). A new prognostic index (CLIPI) for advanced cutaneous lymphoma enables precise patient risk stratification. Blood.

[5] Dobos G., Pohrt A., Ram-Wolff C., Lebbé C., Bouaziz J.-D., Battistella M., Bagot M., de Masson A. (2020). Epidemiology of Cutaneous T-Cell Lymphomas: A Systematic Review and Meta-Analysis of 16,953 Patients. Cancers.

[6] Bradford P.T., Devesa S.S., Anderson W.F., Toro J.R. (2009). Cutaneous lymphoma incidence patterns in the United States: A population-based study of 3884 cases. Blood.

[7] Cheikh El Najjarine K., Kajüter H., Wellmann I., Stang A., Assaf C. (2025). Incidence and Mortality of Cutaneous Lymphomas in Germany. J. Dtsch. Dermatol. Ges..

[8] Latzka J., Assaf C., Bagot M., Cozzio A., Dummer R., Guenova E., Gniadecki R., Hodak E., Jonak C., Klemke C.-D. (2023). EORTC consensus recommendations for the treatment of mycosis fungoides/Sézary syndrome–Update 2023. Eur. J. Cancer.

[9] NCCN Primary Cutaneous Lymphomas, Version 2025. https://www.nccn.org.

[10] Willemze R., Hodak E., Zinzani P., Specht L., Ladetto M. (2018). Primary cutaneous lymphomas: ESMO Clinical Practice Guidelines for diagnosis, treatment and follow-up. Ann. Oncol..

[11] Alberti-Violetti S., Ardigò M., Massone C., Pileri A., Sala R., Teoli M., Grandi V., Quaglino P., Pimpinelli N., Berti E. (2024). Effectiveness and tolerability of chlormethine gel for the management of mycosis fungoides: A multicenter real-life evaluation. Front. Oncol..

[12] Marchi E., Alinari L., Tani M., Stefoni V., Pimpinelli N., Berti E., Pagano L., Bernengo M.G., Zaja F., Rupoli S. (2005). Gemcitabine as frontline treatment for cutaneous T-cell lymphoma. Cancer.

[13] Wollina U., Dummer R., Brockmeyer N.H., Konrad H., Busch J., Kaatz M., Knopf B., Koch H., Hauschild A. (2003). Multicenter study of pegylated liposomal doxorubicin in patients with cutaneous T-cell lymphoma. Cancer.

[14] Hughes C.F.M., Khot A., McCormack C., Lade S., Westerman D.A., Twigger R., Buelens O., Newland K., Tam C., Dickinson M. (2015). Lack of durable disease control with chemotherapy for mycosis fungoides and Sézary syndrome: A comparative study of systemic therapy. Blood.

[15] Quaglino P., Maule M., Prince H.M., Porcu P., Horwitz S., Duvic M., Talpur R., Vermeer M., Bagot M., Guitart J. (2017). Global patterns of care in advanced stage mycosis fungoides/Sezary syndrome: A multicenter retrospective follow-up study from the Cutaneous Lymphoma International Consortium. Ann. Oncol..

[16] Assaf C., Illidge T.M., Waser N., He M., Li T., Zomas A., Bent-Ennakhil N., Little M., Ortiz-Romero P.L., Pimpinelli N. (2023). A Retrospective Chart Review of Treatment Patterns and Overall Survival among a Cohort of Patients with Relapsed/Refractory Mycosis Fungoides in France, Germany, Italy, Spain and the United Kingdom. Cancers.

[17] Francisco J.A., Cerveny C.G., Meyer D.L., Mixan B.J., Klussman K., Chace D.F., Rejniak S.X., Gordon K.A., DeBlanc R., Toki B.E. (2003). cAC10-vcMMAE, an anti-CD30–monomethyl auristatin E conjugate with potent and selective antitumor activity. Blood.

[18] Prince H.M., Kim Y.H., Horwitz S.M., Dummer R., Scarisbrick J., Quaglino P., Zinzani P.L., Wolter P., Sanches J.A., Ortiz-Romero P.L. (2017). Brentuximab vedotin or physician’s choice in CD30-positive cutaneous T-cell lymphoma (ALCANZA): An international, open-label, randomised, phase 3, multicentre trial. Lancet.

[19] Kim Y.H., Prince H.M., Whittaker S., Horwitz S.M., Duvic M., Bechter O., Sanches J.A., Stadler R., Scarisbrick J., Quaglino P. (2021). Response to brentuximab vedotin versus physician’s choice by CD30 expression and large cell transformation status in patients with mycosis fungoides: An ALCANZA sub-analysis. Eur. J. Cancer.

[20] Horwitz S.M., Scarisbrick J.J., Dummer R., Whittaker S., Duvic M., Kim Y.H., Quaglino P., Zinzani P.L., Bechter O., Eradat H. (2021). Randomized phase 3 ALCANZA study of brentuximab vedotin vs physician’s choice in cutaneous T-cell lymphoma: Final data. Blood Adv..

[21] Engelina S., Saggu M., Yoo J., Shah F., Stevens A., Irwin C., Chaganti S., Scarisbrick J. (2019). Brentuximab a novel antibody therapy: Real-world use confirms efficacy and tolerability for CD30-positive cutaneous lymphoma. Br. J. Dermatol..

[22] Papadavid E., Kapniari E., Pappa V., Nikolaou V., Iliakis T., Dalamaga M., Jonak C., Porkert S., Engelina S., Quaglino P. (2021). Multicentric EORTC retrospective study shows efficacy of brentuximab vedotin in patients who have mycosis fungoides and Sézary syndrome with variable CD30 positivity*. Br. J. Dermatol..

[23] Muniesa C., Gallardo F., García-Doval I., Estrach M.T., Combalia A., Morillo-Andújar M., De la Cruz-Vicente F., Machan S., Moya-Martínez C., Rovira R. (2022). Brentuximab vedotin in the treatment of cutaneous T-cell lymphomas: Data from the Spanish Primary Cutaneous Lymphoma Registry. J. Eur. Acad. Dermatol. Venereol..

[24] Barta S.K., Liu N., DerSarkissian M., Chang R., Ye M., Duh M.S., Surinach A., Fanale M., Yu K.S. (2023). Real-World Treatment Patterns and Clinical Outcomes With Brentuximab Vedotin or Other Standard Therapies in Patients With Previously Treated Cutaneous T-Cell Lymphoma in the United States. Clin. Lymphoma Myeloma Leuk..

[25] Olsen E., Vonderheid E., Pimpinelli N., Willemze R., Kim Y., Knobler R., Zackheim H., Duvic M., Estrach T., Lamberg S. (2007). Revisions to the staging and classification of mycosis fungoides and Sézary syndrome: A proposal of the International Society for Cutaneous Lymphomas (ISCL) and the cutaneous lymphoma task force of the European Organization of Research and Treatment of Cancer (EORTC). Blood.

[26] Campbell B.A., Scarisbrick J.J., Kim Y.H., Wilcox R.A., McCormack C., Prince H.M. (2020). Time to Next Treatment as a Meaningful Endpoint for Trials of Primary Cutaneous Lymphoma. Cancers.

[27] Schwab U., Stein H., Gerdes J., Lemke H., Kirchner H., Schaadt M., Diehl V. (1982). Production of a monoclonal antibody specific for Hodgkin and Sternberg–Reed cells of Hodgkin’s disease and a subset of normal lymphoid cells. Nature.

[28] Schwarting R., Gerdes J., Durkop H., Falini B., Pileri S., Stein H. (1989). BER-H2: A new anti-Ki-1 (CD30) monoclonal antibody directed at a formol- resistant epitope. Blood.

[29] Sabattini E., Pizzi M., Tabanelli V., Baldin P., Sacchetti C.S., Agostinelli C., Zinzani P.L., Pileri S.A. (2013). CD30 expression in peripheral T-cell lymphomas. Haematologica.

[30] Karube K., Kakimoto Y., Tonozuka Y., Ohshima K. (2021). The expression of CD30 and its clinico-pathologic significance in peripheral T-cell lymphomas. Expert Rev. Hematol..

[31] Falini B., Pileri S., Pizzolo G., Durkop H., Flenghi L., Stirpe F., Martelli M., Stein H. (1995). CD30 (Ki-1) molecule: A new cytokine receptor of the tumor necrosis factor receptor superfamily as a tool for diagnosis and immunotherapy. Blood.

[32] Wehkamp U., Mitteldorf C., Stendel S., Stranzenbach R., Nicolay J., Wobser M., Weichenthal M., Schneiderbauer R., Klemke C., Hillen U. (2021). Most rare subtypes of cutaneous lymphoma display variable CD30 expression: Analysis of the German Cutaneous Lymphoma Network. Br. J. Dermatol..

[33] Stein H., Falini B. (2025). CD30 as a Target Molecule in the Diagnosis and Therapy of Lymphomas. Am. J. Hematol..

[34] van de Donk N.W.C., Dhimolea E. (2012). Brentuximab vedotin. mAbs.

[35] Jiang Y., Dong S., Wang Y. (2025). Antibody–Drug Conjugates Targeting CD30 in T-Cell Lymphomas: Clinical Progression and Mechanism. Cancers.

[36] Debliquis A., Baseggio L., Bouyer S., Guy J., Garnache-Ottou F., Genevieve F., Mayeur-Rousse C., Letestu R., Chapuis N., Harrivel V. (2020). Multicentric MFI30 study: Standardization of flow cytometry analysis of CD30 expression in non-Hodgkin lymphoma. Cytom. Part B Clin. Cytom..

[37] Mitteldorf C., Kampa F., Ströbel P., Schön M.P., Kempf W. (2022). Intraindividual variability of CD30 expression in mycosis fungoides –implications for diagnostic evaluation and therapy. Histopathology.

[38] Huen A., Rahbar Z., Talpur R., Li S., Hong E., Tetzlaff M., Duvic M., Kim Y. (2018). Updated combined analysis of two phase II studies of brentuximab vedotin in patients with mycosis fungoides and Sézary syndrome. Eur. J. Cancer.

[39] Jagadeesh D., Horwitz S., Bartlett N.L., Kim Y., Jacobsen E., Duvic M., Little M., Trepicchio W., Fenton K., Onsum M. (2022). Response to Brentuximab Vedotin by CD30 Expression in Non-Hodgkin Lymphoma. Oncol..

[40] Shi Y., Li X. (2025). Challenges of CD30 expression and its impact on targeted treatment responses in non-Hodgkin lymphoma: New perspectives for evaluation and validation. Pathol. Res. Pr..

[41] Furudate S., Fujimura T., Kakizaki A., Kambayashi Y., Asano M., Watabe A., Aiba S. (2016). The possible interaction between periostin expressed by cancer stroma and tumor-associated macrophages in developing mycosis fungoides. Exp. Dermatol..

[42] Assaf C., Hwang S.T. (2016). Mac attack: Macrophages as key drivers of cutaneous T-cell lymphoma pathogenesis. Exp. Dermatol..

[43] Prince H.M., Gautam A., Kim Y.H. (2018). Brentuximab vedotin: Targeting CD30 as standard in CTCL. Oncotarget.

[44] Li Z., Guo W., Bai O. (2023). Mechanism of action and therapeutic targeting of CD30 molecule in lymphomas. Front. Oncol..

[45] Hofer V., Maurus K., Houben R., Schrama D., Roth S., Goebeler M., Geissinger E., Rosenwald A., Wobser M. (2021). Treatment of mycosis fungoides with brentuximab vedotin: Assessing CD30 expression by immunohistochemistry and quantitative real-time polymerase chain reaction. J. Cutan. Pathol..

[46] Cieslak C.M., Mitteldorf C., Krömer-Olbrisch T., Kempf W., Stadler R. (2023). QuPath Analysis for CD30+ Cutaneous T-Cell Lymphoma. Am. J. Dermatopathol..

[47] Oymanns M., Daum-Marzian M., Bellm A., Elsayad K., Eich H.T., Assaf C. (2022). Near complete responses to concurrent brentuximab vedotin and ultra-hypofractionated low-dose total skin electron beam radiation in advanced cutaneous T-cell lymphoma. Br. J. Dermatol..

[48] Schummer P., Glatzel C., Schrüfer P., Lawrenz I., Dobos G., Wehkamp U., Hüning S., Stranzenbach R., Nicolay J.P., Goebeler M. (2025). Brentuximab vedotin and radiotherapy for CD30-positive cutaneous T-cell lymphoma—A retrospective multicenter analysis. JDDG J. Dtsch. Dermatol. Ges..

[49] Jo T., Sakai T., Matsuzaka K., Shioya H., Tominaga H., Kaneko Y., Hayashi S., Matsuo M., Taguchi J., Abe K. (2020). Successful Treatment of a Patient with Brentuximab Vedotin-Refractory ALK-Negative Anaplastic Large Cell Lymphoma with Romidepsin. Case Rep. Oncol..

[50] Rozati S., Kim Y.H. (2016). Experimental treatment strategies in primary cutaneous T-cell lymphomas. Curr. Opin. Oncol..

